# Surgical correction of multiple renoduodenal fistulas due to chronic pyelonephritis: a case report and literature review

**DOI:** 10.1093/jscr/rjae051

**Published:** 2024-02-16

**Authors:** Amber L Jones, Lindsey A Braden, Shahab P Hillyer

**Affiliations:** Division of Acute Care and Trauma Surgery, Department of Surgery, Kern Medical Center, 1700 Mount Vernon Avenue, Bakersfield, CA 93306, United States; Division of Acute Care and Trauma Surgery, Department of Surgery, Kern Medical Center, 1700 Mount Vernon Avenue, Bakersfield, CA 93306, United States; Department of Surgery, Harbor-UCLA Medical Center, 1000 West Carson Street, Torrance, CA 90509, United States; Division of Urologic Surgery, Kern Medical Center, Mount Vernon Avenue, Bakersfield, CA 93306, United States

**Keywords:** renal-duodenal fistula, staghorn calculi, chronic pyelonephritis

## Abstract

Renoduodenal fistulas are a rare and uncommon phenomenon that account for ˂1% of those found between the urinary and intestinal tracts. Precipitation of this pathologic tract can be caused by chronic inflammation, necrosis, or ischemia. This case illustrates a 72-year-old man presenting with flank pain discovered to have multiple renoduodenal fistulas and our approach that led to the resolution of his symptoms. We review the pathophysiology, management, and effects of these fistulous tracts on renal function. Patients with staghorn calculi should undergo immediate evaluation for removal of the stone. In cases complicated by fistula formation, need for radical nephrectomy should be investigated and surgical repair should be pursued.

## Introduction

Renoduodenal fistulas are a rare phenomenon that most commonly arise from untreated chronic pyelonephritis, typically precipitated by obstructing renal staghorn calculi. Patients typically experience a combination of fever, leukocytosis, and lower urinary tract symptoms. Because these symptoms are often masked by the underlying disease, its finding is often incidental [1]. Herein, we report a rare case of renoduodenal fistula caused by chronic pyelonephritis secondary to unilateral obstructing staghorn calculi in a 72-year-old man who underwent surgical intervention with an excellent outcome.

## Case presentation

A 72-year-old retired, Caucasian man with a past medical history of nephrolithiasis, and recurrent urinary tract infections presented to an outside hospital with complaints of right flank pain and chills for 3 days. Computed tomography (CT) of the abdomen revealed right renal staghorn calculi, right sided hydronephrosis, and hydroureter. Ureteroscopy revealed a distal right ureteral stricture and several obstructing stones. Consequently, attempts at right ureteral stent placement were unsuccessful, and instead a right-sided nephrostomy tube was placed. Nephrostogram revealed a large, chronic appearing fistula, tracking from the proximal ureter to the duodenum. Total parenteral nutrition (TPN) was initiated, and oral intake was held for 1 week. The need for eventual nephrectomy was discussed with the patient. Due to limitations of scheduling and physician availability, the patient was referred to the author’s facility.

On presentation to our facility, the patient complained of dyspnea on exertion and right arm pain. CT angiography of the chest revealed deep soft tissue inflammation along the left upper extremity peripherally inserted central catheter (PICC) line insertion, extending into the anterior mediastinum. Left upper extremity venous duplex revealed extensive deep vein thrombosis extending from the PICC insertion to the jugular, subclavian, axillary, and brachial veins. Thus, therapeutic Lovenox was started, the PICC line was discontinued, and a nasojejunostomy tube was placed to administer feedings. Nephrostomy-collected urine cultures grew Pseudomonas species, and gram stain revealed gram-negative rods, gram-positive cocci resembling staphylococcus/streptococcus, and budding yeast resembling Candida. Appropriately, IV pantoprazole, pipercillin/tazobactam, vancomycin, and fluconazole were started.

**Figure 1:**
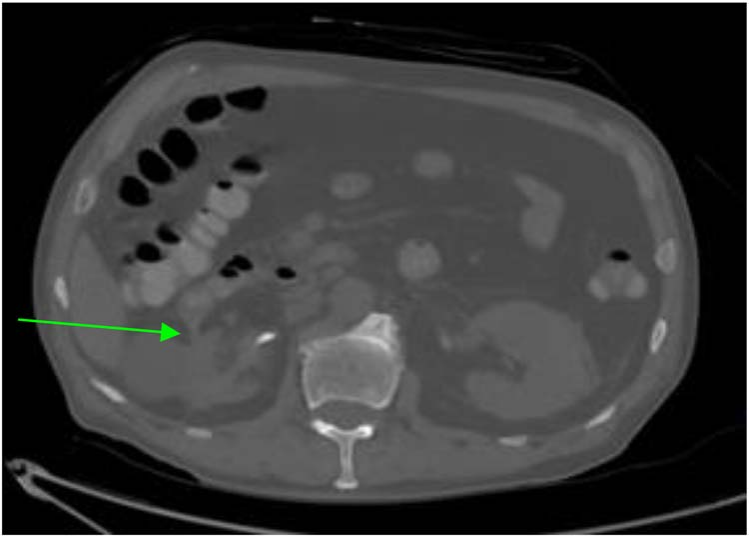
CT abdomen and pelvis revealing a right sided renal-duodenal fistulous connection.

**Figure 2:**
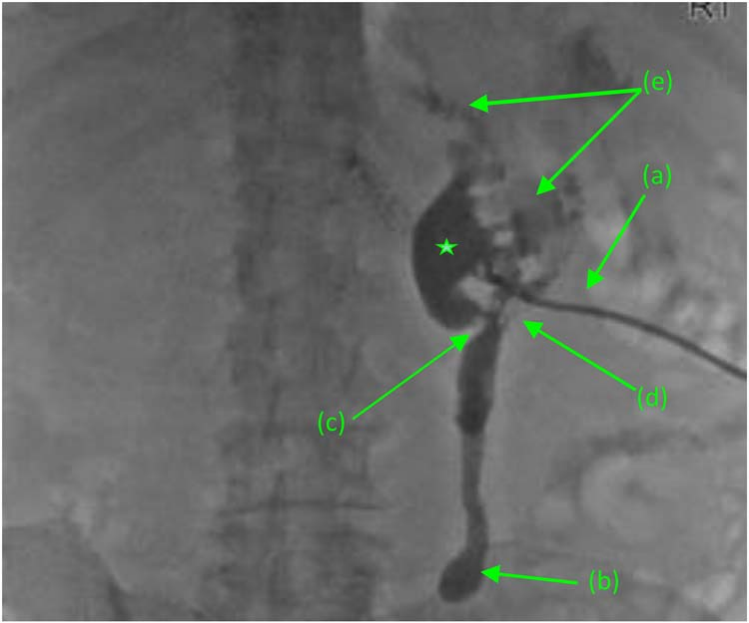
Antegrade pyelogram (prone position) revealing (a) right percutaneous nephrostomy tube (PCN), following the failed ureteral stent placement due to a distal ureteral stricture with subsequent (b) right sided hydroureter and abrupt cutoff. (*) Right sided hydronephrosis, secondary to (c) a second, more proximal ureteral stricture with a (d) frank fistulous connection tracking between the proximal ureter and duodenum. (e) Suspected retrograde leakage of contrast media from the right renal pelvis into the duodenum.

Repeat abdominal CT revealed a fistulous connection between the 3rd portion of the duodenum and the right proximal ureter with oral contrast pooling inferiorly in the mid-distal right ureter (Fig. 1). Nephrostogram confirmed a frank fistulous connection between the proximal ureter and duodenum. Additionally, a second fistula originating from the collecting system superiorly was suspected due to contrast extravasation tracking directly to the 2nd portion of the duodenum (Fig. 2).

**Figure 3:**
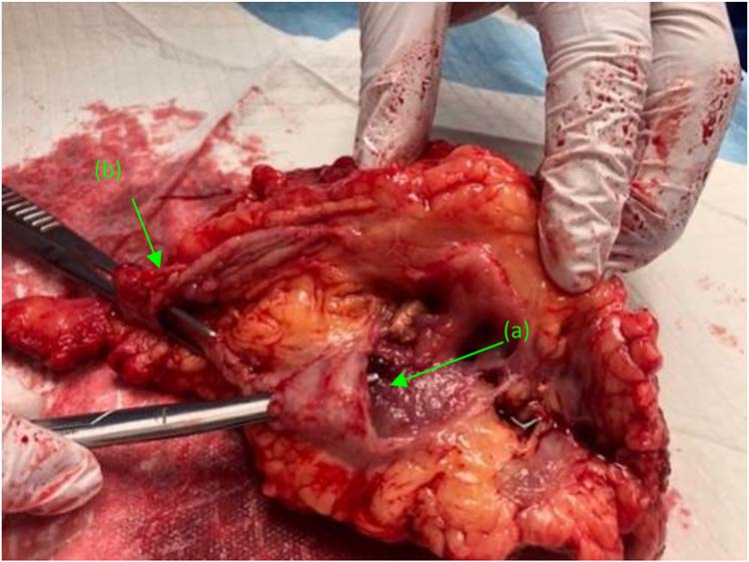
Right kidney, status post nephrectomy, transected at the renal hilum. (a) First fistulous tract exiting the renal pelvis. (b) Second fistulous tract extending from its origin point off the proximal ureter and branching just distal to the ureters entry at the renal pelvis.

Given results of Mercaptiacetyltriglycerine (MAG3) renal scan, revealing a largely non-functioning right kidney (<10%), decision was made to proceed with nephrectomy and duodeno-ureteral fistula repair. A standard posterior nephrectomy approach was utilized, with the initial incision made at the level of the 12th rib, extending to the subcostal margin and cephalad through each layer of muscle from the external oblique to the internal oblique down to the transversalis fascia. The inflamed right ureter was noted just above the iliac artery and isolated using a vessel loop. Two fistulous connections were then identified. The first tract connected the renal pelvis to the second portion of the duodenum. The second fistula connected the proximal ureter to the third portion of the duodenum (Fig. 2). Both tracts were sharply dissected free, tagged with silk ties, and transected. The anterior dissection was extended medially until the right renal vein was identified. The renal hilum was then transected, as were the cephalad attachments (Fig. 3). The right adrenal gland was noted and spared. The kidney was then freed; the specimen was delivered and handed off the field.

**Figure 4:**
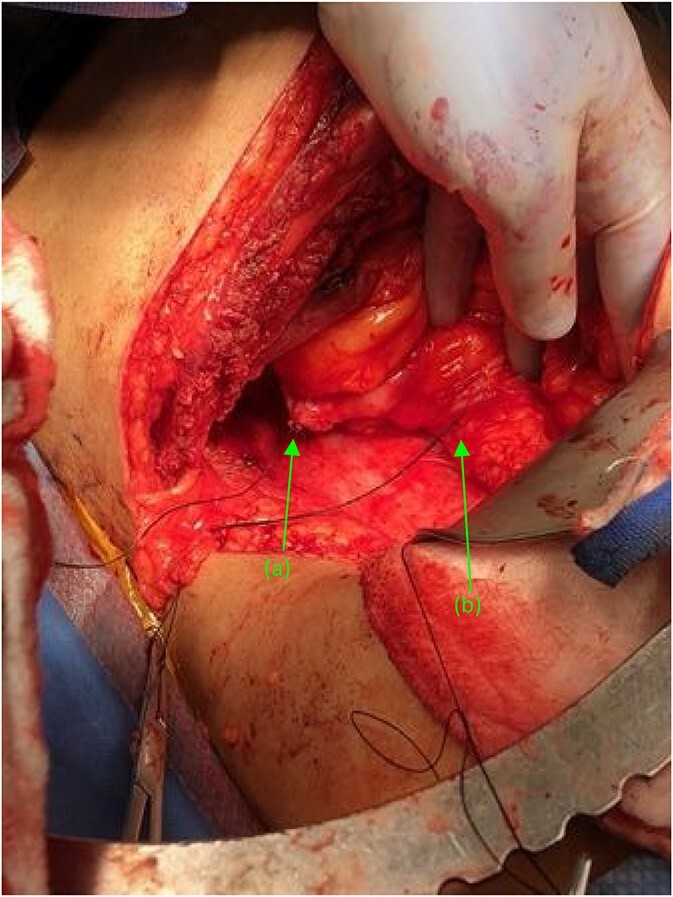
Two suture tagged fistulas status post fistulectomy and debridement to their base, entering the (a) second and (b) third portions of the duodenum, status post nephrectomy.

**Figure 5:**
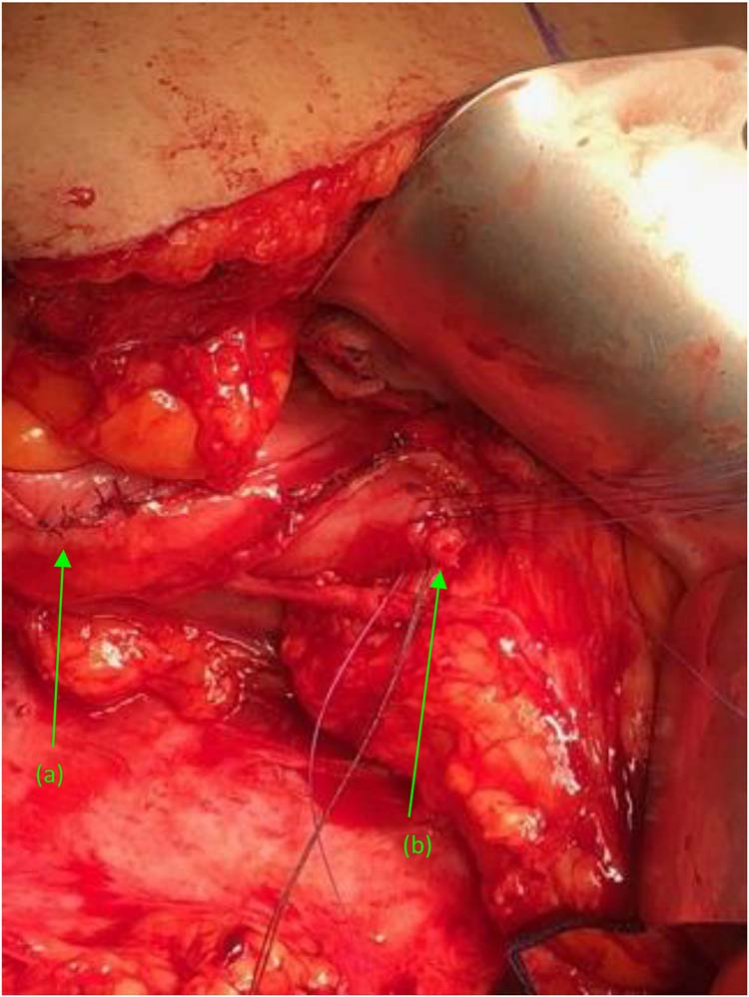
(a) Repaired duodenal defect where the (first) fistula previously entered the second portion of the duodenum, status post fistulectomy and stump closure. (b) Second fistula indicated by suture tags status fistulectomy and trimming to the base of the fistulous tract, near its entry point at the third portion of the duodenum.

Attention was turned back to the fistulous connections at their duodenal entry points (Fig. 4). Both fistulas, were trimmed to their base, debrided, and primarily closed using 3-0 Vicryl sutures in an interrupted fashion. Interrupted 3-0 Vicryl sutures were then used to Lambert the repaired 2nd and 3rd portions of the duodenum (Fig. 5). Acti-seal sealant was sprayed atop the repairs, and Gerota’s was laid atop. Surgicel was then placed in the raw, inflamed posterior space of the fossa and adequate hemostasis was achieved. A 19 French Blake drain was brought through an inferior skin incision, laid in the renal fossa adjacent to the duodenal repairs, and sutured in place. The abdomen was then closed in the standard fashion, with individual suture closure of the fascia, internal and external oblique muscles and skin layers.

The Postoperative course was satisfactory. He was able to resume nasogastric tube feeding on postoperative day (POD) 2. On POD 3, Blake drain output was <50 cc over 24 h. The patient was advanced to oral feedings on POD 4 without increase in the drain output. He recovered well without symptoms of delayed gastric emptying or anastomotic leakage. He was discharged on POD 10 and remained stable during outpatient follow-up visits.

## Discussion

Renoduodenal fistula is an encompassing term that describes any aberrant passage originating from the renal system. It is a rare complication that most commonly occurs in patients with chronic pyelonephritis and often extends from the right kidney to the 2nd and 3rd portions of the duodenum due to the proximity of both structures [2].

The exact mechanism of formation is unclear, although a literature review of other chronic inflammatory conditions, such as Crohn’s disease, suggests that the inflammatory milieu created by chronic renal obstruction, either by nephrolithiasis, or other etiologies including traumatic, iatrogenic, infectious (tuberculosis, roundworm infestation, etc.), or inflammatory injury (IBD, malignancies, etc.), may contribute to tissue erosion and remodeling. However, history as well as initial laboratory evaluation rarely offers clues for the presence of a fistula, as these are non-specific and typical symptoms, such as flank pain, and fever are more consistent with pyelonephritis [2–4]. Physical examination often elicits tenderness and less frequently, a palpable mass. Urinalysis most commonly reveals pyuria and bacteriuria. Polymicrobial infections are possible; however, *Escherichia coli* (*E. coli*) and *Proteus* species remain the predominant isolates [3].

Non-contrast CT is the preferred method in the initial evaluation of pyelonephritis. Although this technique may detect an abnormal connection between the kidney and duodenum, it does not confirm the patency that defines fistulas. Instead, oral or nephrostomy administered contrast can confirm the tract’s presence, after observing its emptying into the nearby enteric tract [3, 5].

Managing a spontaneous renoduodenal fistula involves source control, repairing the defect and radical nephrectomy, as the persistent and chronically undetected insult to the kidney leads to its functional decline [6]. The exact approach, either through surgical or medical intervention, depends on the nature of the disease, patient’s condition, and preference. Few documented cases detailing the exact surgical method of radical nephrectomy and renal-duodenal fistulectomy are available; thus, data remains sparse. In general, the surgeon can opt for transperitoneal or retroperitoneal approaches to access the perirenal area, each with its advantages and disadvantages. The transperitoneal approach offers a larger working space, better maneuverability, and easy orientation to anatomical landmarks; therefore, it is often preferred for laparoscopic exploration. However, reaching the retroperitoneal space, requires significant bowel manipulation and can expose visceral content to potential seeding from ongoing renal infection or malignancy. In contrast, the retroperitoneal route confines the operation to one anatomical space, allows direct access to the targeted organ, and generally requires a shorter operating time [7, 8]. However, the retroperitoneal area is spatially limited, which remains the largest drawback to the approach, and increases the risks of disorientation and potential iatrogenic injury. Given our patient’s fistula location, polymicrobial infection on presentation, and risk of bacterial seeding we believed that open surgery through a retroperitoneal approach offered the best prognosis while minimizing risk of complications. Indeed, our patient recovered well lacking complications and remained without further complaints during his outpatient follow-up course.

Spontaneous closure of a fistula with medical management has been reported in patients with multiple analgesic-induced chronic duodenal ulcers. Similarly, conservative management utilizing long-term broad-spectrum antibiotic or endoscopic clipping method, has also been an effective alternative [7–10]. However, radical nephrectomy followed by primary closure of the fistula is recommended in most patients with extensive renal impairment that serve no functionality.

## Conclusion

Chronically obstructing renal calculi can create a niche for bacterial overgrowth, thereby precipitating pyelonephritis. Without proper treatment, the inflammatory milieu can lead to tissue destruction and remodeling, forming fistular tracts. All patients with staghorn calculi should undergo immediate evaluation, followed by removal of the stone. In cases complicated by fistula precipitation due to chronic pyelonephritis, surgical repair in conjunction with nephrectomy is recommended.

## Data Availability

All data related to the content of this case report is present within the body of the manuscript.
